# Hypoxia After Abdominal and Thigh Liposuction: Pulmonary Embolism or Fat Embolism?

**Published:** 2014-07-17

**Authors:** L. Cohen, R. Engdahl, G. Latrenta

**Affiliations:** Division of Plastic Surgery, New York-Presbyterian Hospital and the Cornell University Medical Center, New York, NY

**Keywords:** fat embolism, abdominal liposuction, pulmonary embolism, fat embolism syndrome, thigh liposuction

## DESCRIPTION

A 58-year-old women with no medical history had new onset chest pain and dyspnea s/p suction-assisted lipectomy of abdomen, bilateral flanks, and medial thighs that day in the office. She presented to the emergency department with new blood-tinged cough and tachypnea with O_2_ saturations in 70s to 80s on room air.

## QUESTIONS

1. **What is the differential diagnosis for the patient's shortness of breath and chest pain?**
2. **How do we diagnose a patient with fat emboli syndrome (FES)?**
3. **How do we manage a patient with the diagnosis of FES?**
4. **Are there methods to reduce fat emboli associated with liposuction?**

## DISCUSSION

The differential diagnosis for acute chest pain and dyspnea includes pulmonary embolism, myocardial infarction, and pneumothorax. After procedures such as fat transfer or liposuction, FES is included in this differential. Fat embolism must be differentiated from venous thromboembolism as the treatment is different and anticoagulation is not used.

Fat emboli syndrome involves blockage of small vessels by fat globules. There are 2 main theories on how this occurs. Physiochemical change in the circulating lipids (chylomicrons) may cause them to clump and form microemboli or trauma causes small veins to rupture and fat to directly enter into the circulation. Regardless of how fat emboli are formed or enter into the vasculature, they have the potential to either shower or lodge in a variety of locations such as lung, brain, kidney, or retina. This can cause a spectrum of symptoms, with local inflammation or endothelial injury in these respective sites furthering injury.

Diagnosis of fat emboli can be difficult because of the nonspecific nature of the initial symptoms. Clinical examination remains at the core of making this diagnosis.^1^ Initial investigations for all with acute respiratory symptoms include rapid clinical evaluation and airway management followed by adjunct tests such as chest radiography, electrocardiogram, and arterial blood gas. The classic FES is defined as the presence of 2 of 3 clinical findings including petechial rash, pulmonary distress, and mental disturbances within the first 48 hours after the inciting event.^2^ Common signs include hypoxia, fever, tachycardia, and tachypnea with bilateral radiographic changes and urinary changes.^3^^,^^4^ Hypoxia is often the first sign^5^ of FES and imaging maybe initially normal or show bilateral lung changes. Scoring systems may be used to aid the diagnosis of FES, such as the Gurd and Wilson^6^ criteria established in the 1970s. This system uses major and minor criteria to help secure the diagnosis of FES.

Treatment is largely supportive. This may include oxygen, continuous positive airway pressure or ventilator support, intravenous fluids, and critical care. Pharmacologic therapy attempting to address fat in the vasculature has yet to be a clear benefit in these patients, and there is no role for therapeutic heparin. Some physicians advocate use of parenteral alcohol as pharmacologic embolism prophylaxis by slowly infusing a liter of 5% alcohol (50 g of ethyl alcohol; a lipase activity inhibitor).^7^ However, many do not use this technique, which is anecdotal in the literature. Physicians should be aware of the potential dilation of the venous system induced by alcohol, which with compensatory hydration may put the patient at higher risk for iatrogenic pulmonary edema.^7^

Specific risk factors for developing FES after liposuction are not well established. Fat manipulation either by liposuction or transfer is likely a clear risk. Other risks may include varicose veins, ultrasonic liquidation of fat cells, cannula size, duration of liposuction, volume of aspirate, number of treated areas, use of postoperative intravenous hydration, and large-volume procedures.^2^^,^^5^^,^^8^ Methods to lower the risk of FES after liposuction include general methods employed to lower the risk and morbidity with liposuction. Important factors include patient selection, careful technique, adequate hydration, staging procedures when necessary, cutting down on operative time, and postoperative monitoring.^2^

## Figures and Tables

**Figure 1:**
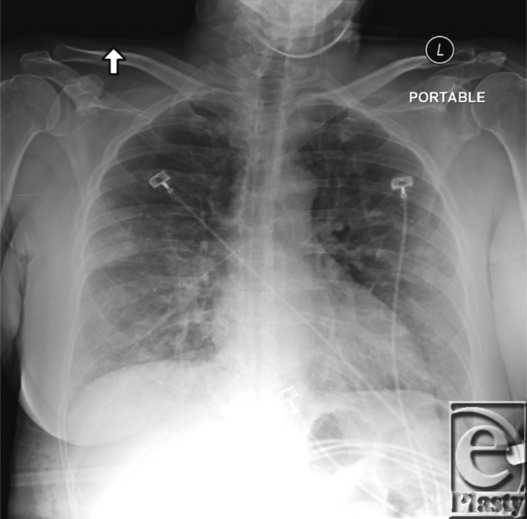
Chest radiograph with bilateral mid and lower lung patchy airspace opacities.

**Figure 2:**
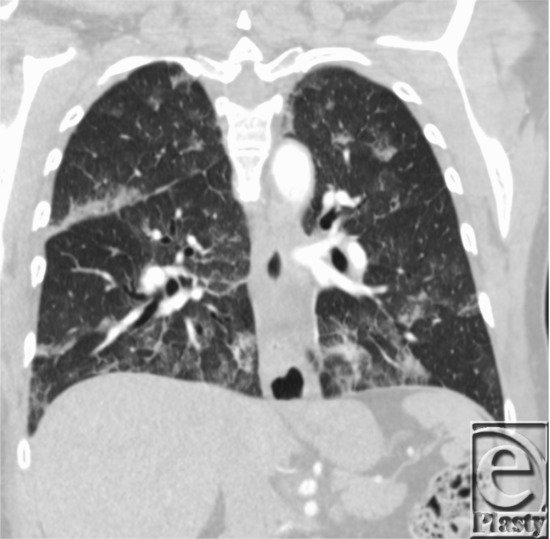
Computed tomographic chest radiograph with patchy diffuse ground-glass change of the pulmonary parenchyma with geographic distribution, interlobular septal thickening, and scattered small nodular opacities.
